# USP39 regulates pyruvate handling in non-small cell lung cancer

**DOI:** 10.1038/s41420-024-02264-0

**Published:** 2024-12-18

**Authors:** Tina Becirovic, Boxi Zhang, Helin Vakifahmetoglu-Norberg, Vitaliy O. Kaminskyy, Elena Kochetkova, Erik Norberg

**Affiliations:** Department of Physiology and Pharmacology, Karolinska Institutet, Biomedicum, Stockholm, Sweden

**Keywords:** Cancer metabolism, Non-small-cell lung cancer, Deubiquitylating enzymes

## Abstract

The ubiquitin-specific peptidase 39 (USP39) belongs to the USP family of cysteine proteases representing the largest group of human deubiquitinases (DUBs). While the oncogenic function of USP39 has been investigated in various cancer types, its roles in non-small cell lung cancer (NSCLC) remain largely unknown. Here, by applying a gene set enrichment analysis (GSEA) on lung adenocarcinoma tissues and metabolite set enrichment analysis (MSEA) on NSCLC cells depleted of USP39, we identified a previously unknown link between USP39 and the metabolism in NSCLC cells. Mechanistically, we uncovered a component of the pyruvate dehydrogenase (PDH) complex, pyruvate dehydrogenase E1 subunit alpha (PDHA), as a target of USP39. We further present that USP39 silencing caused an elevation in Lys^63^ ubiquitination on PDHA and a reduction in the PDH complex activity, the levels of TCA cycle intermediates, mitochondrial respiration, cell proliferation in vitro, and of tumor growth in vivo. Consistently, citrate supplementation restored mitochondrial respiration and cell growth in USP39-depleted cells. Our study elucidates and describes how USP39 regulates pyruvate metabolism through a deubiquitylation process that affects NSCLC tumor growth.

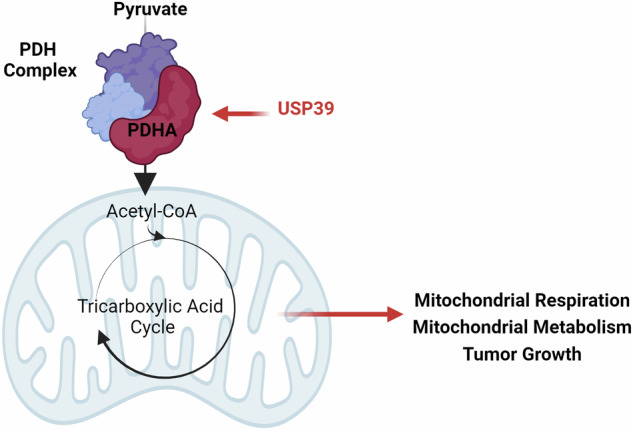

## Introduction

Malignant tissues are characterized by uncontrolled proliferation and require altered metabolic processes to maintain high energy and nutrient supply demands [1]. These metabolic pathways are governed by complex mechanisms and involve the coordination of various signaling molecules. Ongoing discoveries present opportunities for metabolic reprogramming as a promising therapeutic target for cancer-specific changes. Therefore, understanding how metabolic pathways change in cancer cells is crucial for the development of novel treatment strategies [2].

Lung cancer is the most diagnosed cancer worldwide, of which *circa* 85% are non-small cell lung cancer (NSCLC). With about 1.8 million deaths in 2022, it represents the most common cause of cancer-related deaths globally regardless of gender, age, or ethnicity [3]. Despite recent advances in personalized targeted treatments and immunotherapies, the high fatality rate illustrates the urgent need for novel innovative approaches to treat this deadly disease [4].

The activities of metabolic pathways are dynamically adjusted by the coordination of various complex networks of cell-extrinsic and intrinsic signaling processes [5]. The abundance of key metabolic enzymes, which dictates metabolic rates, is controlled by genetic alterations, gene expression, and posttranslational modifications that enhance protein stability. Among the latter, ubiquitin modifications play a crucial role as part of the ubiquitin proteasome system (UPS), which controls protein turnover through degradation [6]. While the process of ubiquitin conjugation involves a coordinated action of ubiquitin-activating (E1), ubiquitin-conjugating (E2), and ubiquitin-ligating (E3) enzymes [7], deubiquitinases (DUBs) function by reversing the action of E3 ligases. By removing ubiquitin molecules from target proteins, DUBs prevent degradation, thereby leading to the stabilization of proteins [8]. Dysregulation of DUBs has been shown to be associated with pathophysiological processes, including tumorigenesis. While DUBs are known to play major roles in various fundamental cellular processes in cancer cells [9], their impact on cancer metabolism remains poorly understood. To date, only a few DUBs, including JOSD2 [10], OTUB2 [11], and USP13 [12], have been shown to directly regulate the stability of metabolic enzymes and thereby modulate metabolic pathway activities. The ubiquitin-specific protease 39 (USP39) has been widely investigated and shown to be critical for multiple cancer-promoting processes including alternative splicing [13], regulation of the cell cycle [14], and DNA damage repair [15]. While its role has been extensively studied in tumor types like endometrial [16], cervical [17], and hepatocellular carcinoma [13, 18–20], its function in NSCLC remains poorly understood [21–24], and no direct deubiquitination of metabolic targets have been reported for USP39 in NSCLC yet.

Pyruvate, which results from the breakdown of glucose in glycolysis, is a crucial metabolite positioned at the crossroads of several essential metabolic pathways. Its key roles include acting as a substrate for the tricarboxylic acid (TCA) cycle and participating in gluconeogenesis, making it essential for cellular energy production and metabolic regulation. The utilization of pyruvate is influenced by numerous factors and is often found to be dysregulated in malignant tissues [25]. The mechanisms underlying this aberrant regulation remain to be further studied.

This study uncovers a novel role of USP39 in NSCLC metabolism. Our data demonstrates that USP39 stabilizes through deubiquitylation the pyruvate dehydrogenase (PDH) complex, a key enzyme complex linking the glycolytic pathway to the TCA cycle in the mitochondria. We further present that the depletion of USP39 leads to degradation of PDH complex components and a reduction in its overall activity, resulting in decreased mitochondrial respiration. Additionally, we show that USP39 deficiency severely impairs cancer cell growth both in vitro and in vivo. Collectively, our findings offer new insights into the role of USP39 in NSCLC metabolism and growth, suggesting a potential vulnerability for developing novel therapeutic strategies.

## Results

### USP39 is associated with NSCLC metabolism

**Fig. 1:**
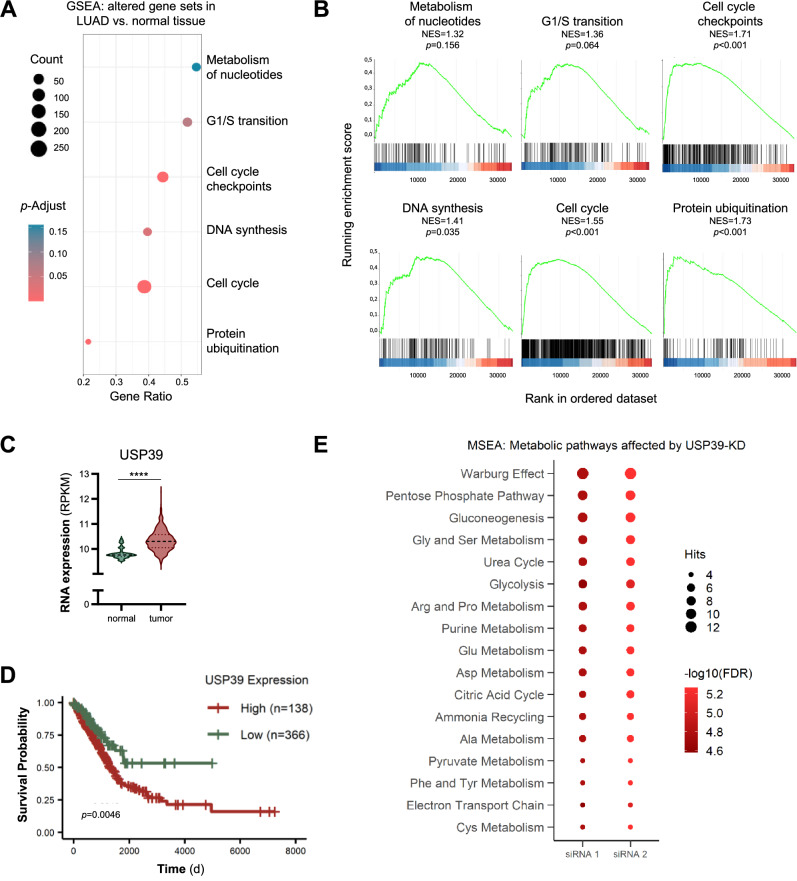
USP39 is associated with NSCLC metabolism. **A** Gene set enrichment analysis (GSEA) of pathways enriched in lung adenocarcinoma tissues (*n* = 535) compared to normal tissues (*n* = 59), RNA expression level data obtained from the TCGA database. **B** Gene sets selectively enriched in lung adenocarcinoma tissues. Normalized enrichment score (NES) and adjusted *p*-value are shown for each gene set. **C** USP39 RNA expression levels in lung adenocarcinoma tissues (*n* = 535) *versus* normal tissues (*n* = 59), data obtained from the TCGA database. **D** Overall survival of lung adenocarcinoma patients with high (*n* = 138) *versus* low (*n* = 366) USP39 expression, data obtained from KM-plotter. **E** Metabolite set enrichment analysis (MSEA) of metabolite level data obtained from USP39-deficient NCI-H1975 cells using CE-TOF/MS (*n* = 3). Significant pathways with at least 4 metabolites altered in that pathway are depicted in the graph.

To start elucidating the role of USP39 in non-small cell lung cancer (NSCLC), we first performed a gene set enrichment analysis (GSEA) with gene expression data from lung adenocarcinoma patients obtained from The Cancer Genome Atlas (TCGA) public database (https://portal.gdc.cancer.gov/) covering the largest group of NSCLC. By comparing cancer patient samples (*n* = 535) to normal tissue samples (*n* = 59), gene sets selectively enriched in lung adenocarcinoma tissues were identified. These included the previously reported cell cycle and DNA synthesis processes, but also uncovered metabolism of nucleotides as a novel pathway (Fig. 1A, B). Beyond the gene sets related to growth and metabolism, the protein ubiquitination gene set was further identified among the top enriched processes.

We examined the TCGA data and found that expression of USP39 was elevated in tumor tissues compared to the non-neoplastic counterparts (Fig. 1C), and that higher expression of USP39 correlated with poor survival (Fig. 1D). As no previous reports indicate a link between USP39 and metabolism, we explored potential effects of USP39 on metabolic pathways by performing loss-of-function studies. After silencing USP39 using two distinct siRNAs (Supplementary Fig. 1A), we compared the metabolite levels to those transfected with a non-targeting control siRNA by mass spectrometry. This analysis revealed a broad effect on multiple metabolic pathways (Supplementary Table 1), including glycolysis, amino acid, purine, and pyruvate metabolism (Fig. 1E), suggesting that USP39 impacts major metabolic pathways.

### USP39 controls the stability and activity of the PDH complex

**Fig. 2:**
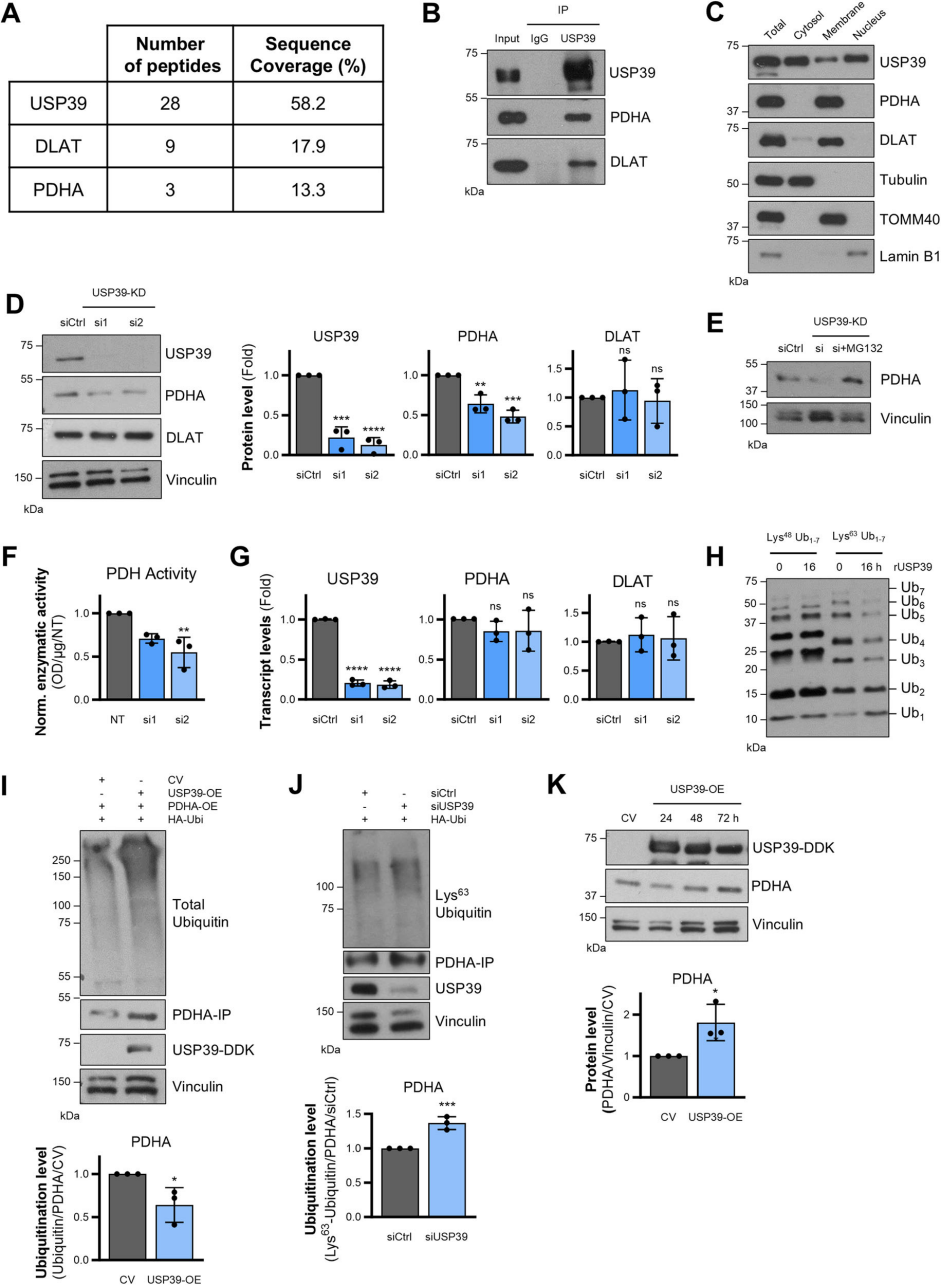
USP39 controls the stability and activity of the PDH complex. **A** LC–MS/MS analysis of immunoconjugates pulled down with USP39 antibody. Number of peptides and coverage of USP39, DLAT, and PDHA. **B** Western blot analysis of immunoconjugates from (**A**). **C** Cellular fractionation of NCI-H1975 cells. Lamin B1, TOMM40 and Tubulin serve as markers for nucleic, membrane, and cytosolic fractions respectively. **D** Protein levels of PDH complex components after 48 h of USP39 siRNA-mediated silencing with quantification (*n* = 3). **E** Protein levels of PDHA after 48 h of USP39 siRNA-mediated silencing and treatment with 5 µM of MG132 for 4 h (*n* = 3). **F** Enzymatic activity of the PDH complex (*n* = 3) after 48 h of USP39 knockdown. **G** mRNA expression levels of PDHA and DLAT upon 48 h of USP39 silencing with siRNAs (*n* = 3). **H** In vitro deubiquitination assay showing the cleavage of Lys^48^ and Lys^63^ U_1-7_ polyubiquitin chains after incubation with recombinant USP39 for 16 h. **I** Ubiquitination level of PDHA in USP39-overexpressing cells and after treatment with MG132 for 4 h (*n* = 3). Quantification of ubiquitination levels were normalized to total PDHA levels and to the control. **J** Lys^63^-ubiquitination level of PDHA in USP39-knockdown cells and after treatment with MG132 for 4 h (*n* = 3). Quantification of Lys^63^-ubiquitination levels were normalized to total PDHA levels and to the control. **K** Protein level of PDHA after 72 h of USP39 overexpression with quantification (*n* = 3). Error bars ± SD. *p ≤ 0.05, **p ≤ 0.01, ***p ≤ 0.001, ****p ≤ 0.0001.

To identify a potential metabolic protein USP39 may target and thereby control its stability, we co-immunoprecipitated USP39 and analyzed its interaction partners by mass spectrometry. LC-MS/MS analysis identified around 500 proteins with more than 3 unique peptides (Supplementary Table 2). Given the metabolic effects observed by USP39 silencing (Fig. 1E), we focused on metabolic enzymes among the identified proteins and found dihydrolipoamide S-acetyltransferase (DLAT) and pyruvate dehydrogenase E1 subunit alpha (PDHA) among the top candidates. In the immunoconjugates, 9 and 3 unique peptides were identified for DLAT and PDHA respectively, covering 17.9 and 13.3% of the total protein sequences (Fig. 2A). To consolidate these findings, the immunoconjugates were additionally analyzed by immunoblotting, confirming the interaction of DLAT and PDHA with USP39 (Fig. 2B). Both proteins are components of the pyruvate dehydrogenase (PDH) complex, suggesting that USP39 may control the stability and activity of this complex. Given that the PDH complex is located in the mitochondria and USP39 is typically known to reside in the nucleus [20, 26, 27], cellular fractionation experiments were conducted to determine their subcellular locations and assess the potential for physical interaction. While the components of the PDH complex were solely found in the membrane fraction containing mitochondria, USP39 was found in all fractions (Fig. 2C). This supports our findings in Fig. 2A, where USP39 and PDH complex components were found to interact. To explore whether USP39 controls the stability of the PDH complex components, siRNA-mediated knockdown of USP39 was performed and the protein levels of the PDH complex components were measured using immunoblotting. Specifically, the protein levels of PDHA were reduced, while DLAT was unaffected (Fig. 2D). Knockdown of other DUBs, namely PSMD14 and OTUB1, did not reduce PDHA protein levels (Supplementary Fig. 1B). The reduced PDHA levels in USP39-knockdown cells could be rescued by treatment with MG132, a proteasome inhibitor, indicating a UPS-related mechanism (Fig. 2E). The decrease in PDHA levels was sufficient to reduce the activity of the PDH complex by approximately 40–60% (Fig. 2F). Silencing of USP39 did not cause a significant change in mRNA expression levels of PDHA (Fig. 2G).

Considering that USP39 silencing reduced PDHA protein levels, we next determined whether USP39 could deubiquitinate PDHA. A prerequisite for USP39 to perform such function is its ability to deubiquitinate ubiquitin moieties. Recombinant USP39 was indeed capable of cleaving Lys^63^ linked polyubiquitin chains (Fig. 2H). The ectopic expression of USP39, under conditions where MG132 was present to prevent proteasomal degradation, led to a more efficient pulldown of PDHA and reduced the level of PDHA ubiquitination (Fig. 2I). Conversely, USP39-deficiency increased the levels of PDHA Lys^63^-ubiquitination levels (Fig. 2J). The increased immunoprecipitation of PDHA (Fig. 2I, second lane) indicated that overexpression of USP39 could elevate the protein level of its target by removing ubiquitin and increasing its stability. Accordingly, USP39 overexpression was found to elevate PDHA protein levels by about 60% (Fig. 2K). Combined, these data reveal a novel function of USP39 to control the stability, ubiquitination, and activity of the PDH complex by identifying PDHA as a novel USP39 target.

### USP39 maintains the supply of pyruvate to the TCA cycle in mitochondria

**Fig. 3:**
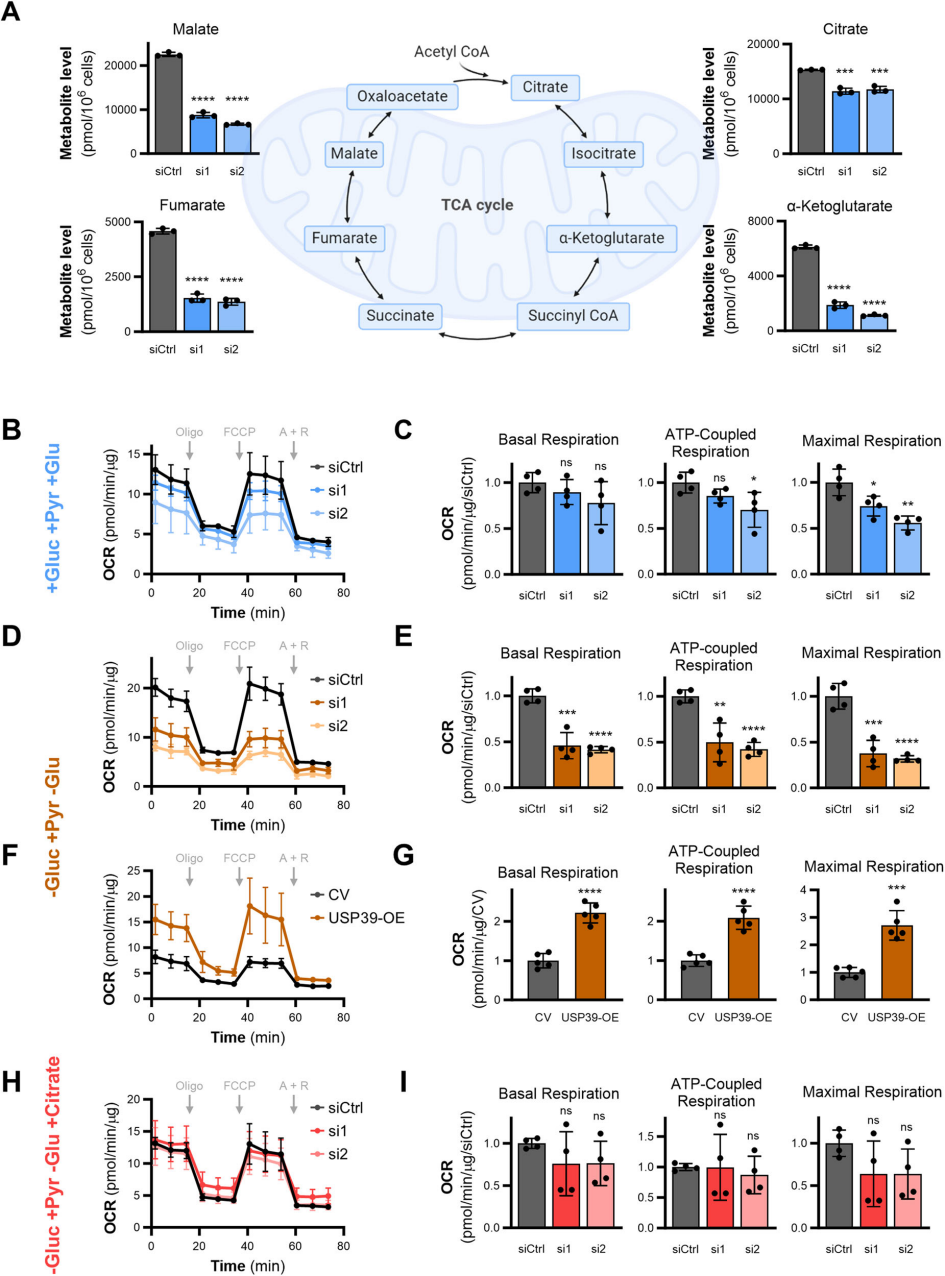
USP39 maintains the supply of pyruvate to the TCA cycle in mitochondria. **A** Metabolite levels of TCA cycle intermediates upon 48 h of USP39 depletion measured by CE-TOF/MS. **B** Oxygen consumption rate (OCR) in USP39 knockdown cells with medium containing 10 mM glucose, 2 mM glutamine, and 1 mM pyruvate. **C** Mitochondrial parameters calculated from (**B**) (*n* = 3). **D** OCR from USP39 knockdown cells supplied with medium containing only 2 mM pyruvate. **E** Mitochondrial parameters calculated from (**D**) (*n* = 3). **F** OCR from USP39 overexpressing cells supplied with medium containing only 2 mM pyruvate. **G** Mitochondrial parameters calculated from (**F**) (*n* = 3). **H** OCR measured in USP39 knockdown cells with medium containing 2 mM pyruvate and 200 mM citrate. **I** Mitochondrial parameters calculated from (**H**) (*n* = 3). Error bars ± SD. *p ≤ 0.05, **p ≤ 0.01, ***p ≤ 0.001, ****p ≤ 0.0001.

The PDH complex converts pyruvate into acetyl-coenzyme A (acetyl-CoA), which provides the acetyl group needed to produce citrate in the TCA cycle. The MSEA analysis of USP39-depleted cells highlighted pyruvate metabolism and the TCA cycle to be significantly altered (Fig. 1E). Therefore, metabolite levels in USP39-depleted cells were analyzed by mass spectrometry-based metabolomics to investigate changes in TCA cycle intermediates. USP39 knockdown resulted in significant decrease of the levels of citrate, α-ketoglutarate, fumarate and malate (Fig. 3A), indicating a dampening of mitochondrial respiration. However, assessment of the oxygen consumption rate (OCR) using an Extracellular Flux Analyzer did not show robust effects on mitochondrial respiration upon USP39 silencing (Fig. 3B, C). This measurement was performed using medium containing glucose, glutamine, and pyruvate, giving the cells multiple different substrates to feed into the TCA cycle. Therefore, we prompted to analyze respiration using medium containing pyruvate as the only fuel, where the conversion of pyruvate to acetyl-CoA by the PDH complex cannot be bypassed by the conversion of glutamine to α-ketoglutarate. Indeed, USP39 silencing caused a significant suppression of OCR in pyruvate-only medium (Fig. 3D, E), and accordingly USP39 overexpression stimulated mitochondrial respiration (Fig. 3F, G). To further challenge whether USP39 is key to controlling the pyruvate-to-acetyl-CoA conversion, OCR was measured in USP39-depleted cells supplemented with citrate, which is a TCA intermediate directly downstream of acetyl-CoA. Mitochondrial respiration may be blunted under such conditions since the PDH complex would not be needed to fuel the TCA cycle. In fact, citrate supplementation to USP39-depleted cells restored the OCR rates (Fig. 3H, I). Altogether, these data show that USP39 affects mitochondrial respiration by regulating the conversion of pyruvate to acetyl-CoA.

### USP39 is required for NSCLC tumor growth

**Fig. 4:**
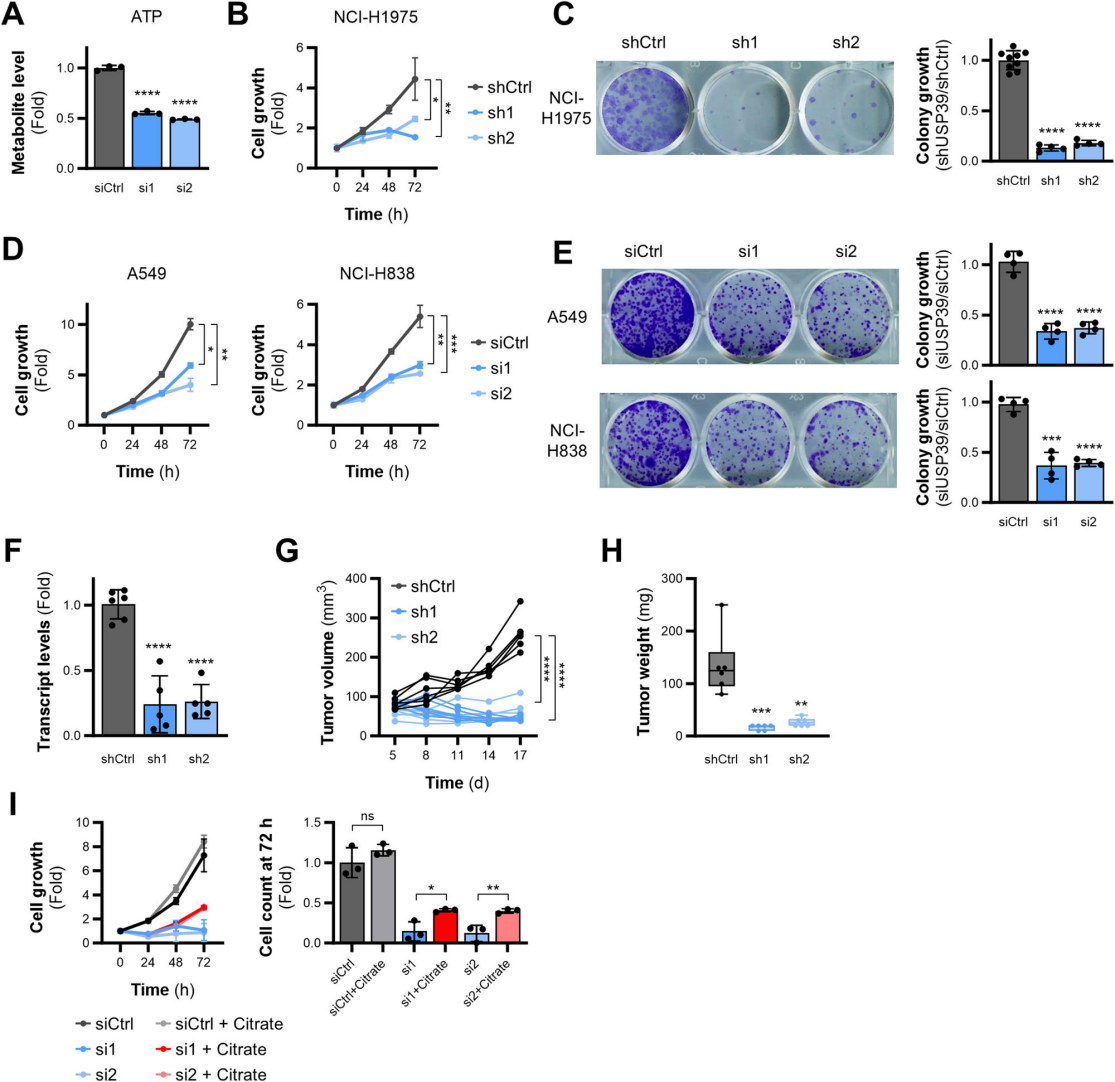
USP39 is required for NSCLC tumor growth. **A** ATP levels upon 48 h of USP39 depletion measured by CE-TOF/MS (*n* = 3). **B** Cell growth of shRNA-expressing USP39 knockdown NCI-H1975 cell lines assessed over 72 h after induction with 20 ng/mL doxycycline for 48 h (*n* = 3). **C** Clonogenic assay with cells from (B), quantification in bar graph (*n* = 3). **D** Cell growth in A549 and NCI-H838 cells upon siRNA-mediated USP39 knockdown. **E** Clonogenic assay with cells from (**D**), quantification in bar graphs (*n* = 3). **F** Knockdown efficiency in xenograft tumors from doxycycline-inducible shRNA-expressing NCI-H1975 cells in mice (*n* = 6 per group). **G** Individual tumor growth of xenograft tumors from USP39-depleted cells measured over 17 days. Statistics were calculated from the averages of each group on day 17. **H** Final weight of tumors from (**E**). **I** Cell growth of NCI-H1975 cells upon siRNA-mediated USP39 knockdown with and without supplementation of 200 mM sodium citrate. Bar graphs show the cell counts after 72 h. Error bars ± SD. *p ≤ 0.05, **p ≤ 0.01, ***p ≤ 0.001, ****p ≤ 0.0001.

To investigate whether the observed effects on mitochondrial respiration upon USP39 depletion affected the growth of NSCLC cells both in vitro and in vivo, doxycycline-inducible NCI-H1975 cell lines expressing either a non-targeting control or USP39 shRNAs were generated (Supplementary Fig. 1C). First, cell growth and clonogenicity were analyzed in vitro. USP39 silencing significantly decreased ATP levels (Fig. 4A), inhibited both cell growth measured over 72 h (Fig. 4B), as well as long-term clonogenic growth (Fig. 4C). A similar effect on cell growth and clonogenicity was observed in two further NSCLC cell lines, namely A549 and NCI-H838 (Fig. 4D, E). Next, to investigate the effects of USP39 suppression on tumor growth, NCI-H1975 cells expressing USP39-targeting shRNA constructs were injected into immunodeficient mice. The mice were provided drinking water with doxycycline to ensure stable knockdown of USP39 (Fig. 4F). Constant suppression of USP39 resulted in major impairment of tumor growth (Fig. 4G) and tumor weights (Fig. 4H).

Considering that citrate supplementation restored the metabolic effects displayed by USP39 deficiency, the next step was to examine its impact on cell growth. USP39 knockdown cells were supplied with citrate and their growth was monitored for the subsequent 72 h. Indeed, citrate supplementation partly restored the growth of USP39-silenced cells (Fig. 4I).

Taken together, our data show that USP39 regulated pyruvate handling and is crucial for cell proliferation in vitro and tumor growth in vivo.

## Discussion

Cancer cells enhance their metabolism to support rapid and uncontrolled proliferation and expansion. Increasing the stability of metabolic enzymes is a fast mechanism to enhance the activity of metabolic pathways. We have previously demonstrated that DUBs can promote the stabilization of metabolic enzymes in cancer [10, 28]. Our current findings provide another novel example of how DUBs can act as regulators of cancer metabolism.

The DUB USP39 is known for its role in regulating alternative splicing and has been reported to promote carcinogenesis and cancer progression through deubiquitination [13, 29, 30]. Although its deubiquitinating activity was initially debated, recent studies have revealed that USP39 can indeed deubiquitinate proteins such as FOXM1 [31], CHK2 [15], and STAT1 [32]. Specifically, USP39 promotes breast cancer cell proliferation and tumor growth by deubiquitinating and stabilizing the transcription factor FOXM1 [31]. Additionally, USP39 stabilizes Cyclin B1 by cleaving polyubiquitin chains, thereby promoting glioma progression [33]. USP39 is frequently overexpressed in various cancer tissues compared to the corresponding non-neoplastic tissues [24, 29–31]. Both as an alternative splicing regulator and as a DUB, USP39 has been shown to promote cancer progression. USP39 has been shown to be able to indirectly affect glutamine metabolism via the mitochondrial ribosomal protein 35 L [22]. However, until now, no metabolic enzyme as a direct target of USP39-mediated deubiquitination has been identified that changes cancer metabolism.

In this study, we uncovered a link between USP39 and NSCLC metabolism through PDHA, a component of the PDH complex. The PDH complex is regulated by a family of four pyruvate dehydrogenase kinases (PDK1-4), which phosphorylate serine residues on the PDHA subunit, inhibiting the activity of the entire complex [34]. In addition, the PDH complex can be downregulated through acetylation of PDHA [35]. Despite the conversion of pyruvate to acetyl-CoA requiring the coordinated activity of its subunits PDHA, DLAT, and DLD, it is the PDHA subunit that is regulated by both phosphorylation-dephosphorylation and acetylation-deacetylation events. The mechanisms that regulate the stability of the PDH complex are less studied, however, it was shown that PDHA is ubiquitinated by the E3 ubiquitin ligase UBE3A, resulting in proteasome-mediated degradation [36]. Our data show that USP39 promotes PDHA stability through a deubiquitination-mediated process. Given its interaction with PDHA, this interaction may act as a competitive binding that prevents ubiquitination, similar to the stabilization mechanism by USP39 described for FOXM1 [31].

Currently, six known routes convert pyruvate into different metabolites. The most studied are the conversion of pyruvate to lactate by the lactate dehydrogenase, to acetyl-CoA by the PDH complex, and to oxaloacetate by the pyruvate carboxylase (PC). Additionally, pyruvate can be converted to alanine, malate, and acetate [37]. Both the PDH complex and PC appear to be crucial in promoting certain types of cancer by sustaining the TCA cycle. For example, Kras-driven NSCLC tumor formation and growth are completely abrogated by PDHA or PC knockout [38]. While oxaloacetate derived from PC contributes to several non-mitochondrial metabolic pathways, such as gluconeogenesis, disruption of the TCA cycle steady-state metabolome is more commonly described as a consequence of PDH complex inhibition [37]. Our study presents that the decrease of PDH complex activity after USP39 depletion results in a drastic decrease of TCA cycle intermediates, which is consistent with other studies that show a decrease of TCA cycle metabolites and carbon flux as a crucial consequence of direct PDHA silencing [38, 39].

Despite NSCLC being heavily reliant on glycolysis [40], tumors benefit greatly from upregulating mitochondrial metabolism as well. TCA cycle metabolites can promote malignant transformation and cancer progression in different ways. Fumarate accumulation can promote cancer progression through HIF upregulation [41, 42]. In tumors with mutated succinate dehydrogenase, succinate can get excreted from the cells and enhance the migratory capacities of neighboring cells and therefore metastasis [43]. Finally, excessively accumulated citrate gets exported from the mitochondria to the cytosol, where it is converted into acetyl-CoA and supplies fatty acid synthesis [44]. Some tumor types are even capable of reversing the TCA cycle flux to preserve the levels of cytoplasmic citrate needed to sustain lipogenesis [45].

Maintaining stable production of TCA cycle metabolites appears to be a crucial factor in cancer development and progression. We have shown that the DUB USP39 acts as a regulator of the PDHA subunit of the PDH complex, governs pyruvate conversion to the TCA cycle, and USP39 depletion has a tremendous effect on the TCA cycle, mitochondrial respiration, cell and tumor growth. Thus, our data propose USP39 as an essential regulator of pyruvate handling in NSCLC.

## Materials and methods

### Cell culture

The NSCLC cell lines NCI-H1975, A549, and NCI-H838 were cultured in RPMI-1640 medium (Sigma-Aldrich, St. Louis, MO, USA) with 10% heat-inactivated fetal bovine serum (FBS, Gibco, Waltham, MA, USA), 2 mM l-glutamine (Sigma-Aldrich), and 100 U/mL penicillin/streptomycin (Sigma-Aldrich). The stable inducible shRNA-expressing NCI-H1975 cell lines were grown in the same medium, except for the usage of Tet-system-approved FBS (Gibco). For the lentiviral production, the human embryonic kidney cell line HEK293T was cultured in DMEM medium (Gibco) with 10% FBS, 2 mM L-glutamine, 100 U/mL penicillin/streptomycin, 1% non-essential amino acids (Sigma-Aldrich), and 100 µM sodium pyruvate (Sigma-Aldrich). All cell lines were kept in a logarithmic growth phase in a humidified incubator with 5% CO_2_. The absence of mycoplasma contaminations was checked frequently with PCR-based testing.

### Analysis of primary NSCLC data

The TCGAbiolink package [46] was used to retrieve the counts and FPKM data of lung adenocarcinoma from the TCGA database. The DESeq2 program was utilized to normalize and evaluate the counts to perform a gene expression comparison between the tumor and the normal tissue. Next, to identify the enriched pathways, the gene set enrichment analysis (GSEA) was carried out using the clusterProfiler package [47].

The RNA expression levels of USP39 were retrieved from the downloaded TCGA data and compared between normal lung tissues (*n* = 59) and lung adenocarcinoma tissues (*n* = 535). Results were visualized as violin plots using GraphPad Prism.

The analysis of overall survival was based on data downloaded from the KM-plotter database [48]. Using RNA expression data (*n* = 504), patients were divided into USP39-high *versus* USP39-low expressing patients. The cutoff was determined by the performance of all possible cutoff values between the lower and upper quantiles. Data was visualized using the survminer package in R [49].

### siRNA-mediated knockdown

The cells were transfected using the Interferin transfection reagent (Polyplus transfection, New York, NY, USA) following the manufacturer’s instructions. USP39 knockdown efficiencies were determined by immunoblotting or qPCR 48 h post transfection. A non-targeting siRNA served as control. The following siRNAs were used: NT Control Pool (D-001810-10, Dharmacon), USP39 siRNA#1 (J-006087-05, Dharmacon), USP39 siRNA#2 (J-006087-07, Dharmacon).

### Metabolome analysis

For the measurement of intracellular metabolite levels, metabolites were extracted from cells as previously described [50], and analyzed using Capillary-Electrophoresis Time of Flight Mass Spectrometer (CE-TOF/MS, Human Metabolome Technologies, Boston, MA, USA). Briefly, NCI-H1975 cells were transfected with siRNAs targeting USP39 or a non-targeting control. After 48 h, cells were washed with 5% mannitol (Sigma-Aldrich) solution, and the metabolites extracted using methanol (Sigma-Aldrich) containing 10 µM Internal Standard Solution (Human Metabolome Technologies). The extracted metabolites were cleared of large cell residues via centrifugation at 2 300 *×* *g* at 4 °C for 5 min and the supernatant was filtered through a Millipore 5-kDa cutoff filter at 9 100 *×* *g* at 4 °C for 2 h.

### Metabolite Set Enrichment Analysis (MSEA)

The Metabolite Set Enrichment Analysis (MSEA) was performed using the MetaboAnalystR package in R [51]. Visualized were the pathways that were significantly altered by knockdown of both independent siRNAs and had at least 4 hits among the measured metabolites.

### Co-immunoprecipitation

The method for co-immunoprecipitation was based on a previously established protocol [28]. Briefly, NCI-H1975 cells were lysed on ice for 15 min with the lysis buffer (1% digitonin, 150 mM NaCl, 25 mM Tris, pH 7.5) supplemented with protease inhibitors (Roche Diagnostics, Risch-Rotkreutz, Switzerland), and protein concentrations determined using the Pierce BCA Protein Assay kit (Thermo Fisher Scientific, Waltham, MA, USA). The Dynabeads Protein A were conjugated with either the isotype antibody control or the USP39 antibody (1.5 µg each) in the antibody-binding buffer (2% BSA in PBS with 0.02% TWEEN® 20) for 4 h at 4 °C on a rotating platform. Cell lysates were incubated with the conjugated antibody-bead complexes overnight. The beads were washed four times with PBS. Finally, 10% of immunoconjugates were used for immunoblotting and the remaining part (about 0.6 mg of initial total protein lysate) was sent for LC-MS/MS analysis.

### Mass spectrometric analysis of immunoconjugates

Immunoconjugates were digested with trypsin on magnetic beads. The resulting peptides were purified and resuspended in 2% acetonitrile (ACN) and 0.1% formic acid (FA) to a final volume of 15 µL. For Nano-flow LC-MS/MS analysis, 2 µL of each sample were injected into an Ultimate 3000 UHPLC system (Thermo Fisher Scientific) connected to a Q Exactive™ HF hybrid Quadrupole-Orbitrap™ mass spectrometer (Thermo Fisher Scientific). Peptides were separated on a 50 cm heated (55 °C) C-18 Easy-Spray™ column (Thermo Fisher Scientific) using a gradient of 4-36% B over 120 minutes at 300 nL/min (solvent A: 2% ACN, 0.1% FA; solvent B: 98% ACN, 0.1% FA). Survey mass spectra were acquired at a resolution of 120,000 (m/z range 350-600). MS/MS data for the 17 most intense precursors were obtained at a resolution of 30,000 using higher-energy collisional dissociation (HCD) with 28% normalized collision energy. MS raw data files were converted to Mascot Generic Format (mgf) using the in-house Raw2mgf program. The mgf files were searched against the SwissProt HUMAN database with the Mascot Server search engine (v2.5.1, MatrixScience Ltd., London, UK), allowing up to two missed trypsin cleavage sites, with mass tolerances of 10 ppm for precursors and 0.02 Da for HCD fragments. Fixed modification was set to cysteine carbamidomethylation, while dynamic modifications included deamination of asparagine and glutamine, and oxidation of methionine. Proteins with at least 3 unique peptides detected in USP39 conjugates but not more than 1 in controls were identified as potential interaction partners.

### Immunoblotting

Cell lysates were prepared in cOmplete™ Lysis-M buffer (Roche Applied Science, Mannheim, Germany) with protease inhibitors, and protein concentrations were determined using the Pierce BCA Protein Assay kit (Thermo Fisher Scientific). Proteins were resolved via SDS-PAGE and transferred onto nitrocellulose membranes, which were probed with primary antibodies (diluted in PBS with 1% BSA and 0.1% NaN_3_) overnight at 4 °C. Secondary HRP-conjugated antibodies (goat anti-mouse or goat anti-rabbit, Thermo Fisher Scientific) were diluted in PBS with 2.5% non-fat dairy milk and 0.05% TWEEN® 20. Protein bands were quantified using ImageJ. The following antibodies were used: DDK (TA50011-100, OriGene, Rockville, MD, USA), DLAT (68303-1-Ig, ProteinTech Group, Chicago, IL, USA), Lamin B1 (12586S, Cell Signaling Technology, Danvers, MS, USA), PDHA (3205S, Cell Signalling), TOMM40 (sc-11414, Santa Cruz Biotech, Dallas, TX, USA), Tubulin (T8203, Sigma-Aldrich, St. Louis, MO, USA), Ubiquitin (3933S, Cell Signalling), Lys^63^-Ubiquitin (5621S, Cell Signalling), USP39 (23865-1-AP, ProteinTech Group), Vinculin (ab129002, Abcam, Cambridge, UK).

### Real-time quantitative PCR (qPCR)

Total RNA was isolated from the cells using the PureLink™ RNA Mini Kit (Invitrogen, Thermo Fisher Scientific) and treated with DNase (Thermo Fisher Scientific). To isolate RNA from tumor tissues, the RNeasy Mini Kit (QIAGEN, Hilden, Germany) was used. The isolated RNA was transcribed to cDNA using the iScript cDNA synthesis kit (BioRad, Hercules, CA, USA). The real-time quantitative PCR (qPCR) was performed with Maxima qPCR SYBR green master mix (Thermo Fisher Scientific) and run on the StepOnePlus™ Real-Time PCR System (Applied Biosystems, Waltham, MA, USA). All kits were executed using the manufacturers’ instructions. The relative mRNA expression was calculated with the ΔΔCT method with Tubulin as the housekeeping gene.

The following primers were used:

DLAT fw 5′-ACTCCCCAGCCTTTAGCTC-3′

DLAT rv 5′-CAATCCCTTTCTCTACTGCCAAC-3′

PDHA fw 5′-GAGTCAGTGCTTCAAGCCAACAG-3′

PDHA rv 5′-GACACGAGCGTCACCTCCATA-3′

Tubulin fw 5′-TCTACCTCCCTCACTCAGCT-3′

Tubulin rv 5′-CCAGAGTCAGGGGTGTTCAT-3′

USP39 fw 5′-GTGTCAGTTCGTCTTGCTCAGC-3′

USP39 rv 5′-GCTGTAACGACCCACATCCTGA-3′

### Sub-cellular fractionation

NCI-H1975 cells were seeded onto 60 mm^2^, then washed twice with PBS, and collected using cell scrapers. The cell suspension was equally divided between two tubes to prepare total lysate and the cellular fractions. The total lysates were prepared by lysing the cells for 15 min on ice in RIPA buffer, supplemented with cOmplete^TM^ protease and phosphatase inhibitor cocktails. The cytosolic fraction was prepared by lysing cells in buffer A (150 mM NaCl, 50 mM Hepes, pH 7.4, 0.02% digitonin, cOmplete^TM^ protease and phosphatase inhibitor cocktails) and incubated for 5 min at room temperature. The obtained extract was centrifuged at 7000 *×* *g* for 5 min and the supernatant was collected as the cytosolic fraction. Then, the pellets were washed 3 times with PBS and the membrane fraction containing mitochondria was extracted using buffer B (150 mM NaCl, 50 mM Hepes, pH 7.4, 0.5% Igepal, cOmplete^TM^ protease and phosphatase inhibitor cocktails) for 15 min on ice. After centrifugation (7000 × *g*, 5 min), the supernatant was collected as the fraction containing mitochondria. After 3 times washing with PBS the nuclear fraction was extracted using buffer C (RIPA lysis buffer supplemented with cOmplete^TM^ protease and phosphatase inhibitor cocktails). TOMM40 served as a marker for the membrane fraction, Lamin B1 for the nuclear fraction, and Tubulin for the cytosolic fraction.

### In vitro deubiquitination assay

For the in vitro ubiquitination assay, recombinant USP39 was purchased from Antibodies Online (ABIN2735114) and polyubiquitin chains were purchased from Boston Biochem. Recombinant USP39 was diluted with assay buffer (40 mM Tris-HCl, pH 7.5, 5 mM DTT, 0.005% BSA) and activated for 10 min at 30 °C. Next, 1 µg of protein was mixed with 200 ng of either Lys^48^ or Lys^63^ polyubiquitin (Ubi_1_–Ubi_7_) chains and incubated at 30 °C for 16 h. The reactions then were stopped by adding SDS sample buffer (Bio-Rad), supplemented with 100 mM DTT. Samples were subjected to immunoblotting with antibodies against ubiquitin.

### Ubiquitination assay

For the detection of ubiquitinated proteins, NCI-H1975 cells were co-transfected with plasmids for the overexpression of PDHA (OriGene, RC201831), HA-ubiquitin (gift from Edward Yeh (Addgene plasmid # 18712; http://n2t.net/addgene:18712 ; RRID:Addgene_18712) [52]), and USP39 (OriGene, RC209551) or a control vector (pCB6+) using the ViaFect transfection reagent (Promega, Madison, WI, USA). For the detection of Lys^63^-ubiquitination levels in USP39-deficient cells, NCI-H1975 cells were transfected with siRNAs targeting USP39 or a non-targeting siRNA control 24 h prior to transfection with the HA-ubiquitin plasmid. After 24 h, cells were treated with 5 µM of the proteasomal inhibitor MG132 (Selleckchem, Houston, TX, USA) for 4 h prior harvesting. Cell lysates were immunoprecipitated with the antibodies targeting PDHA (ProteinTech, 66119-1-Ig). Immunoblotting of the immunoconjugates with anti-Ubiquitin and anti-PDHA antibodies was performed to assess the ubiquitination status of PDHA. Ubiquitination levels were quantified using ImageJ.

### PDH activity assay

The measurement of PDH complex enzymatic activity was performed using the Abcam Pyruvate Dehydrogenase Kit (ab287837) according to manufacturer’s instruction. After 48 h of USP39 knockdown, 1 × 10^6^ cells were collected in ice-cold assay buffer and lysed. Lysates were incubated with the reaction mix for 15 min at 37 °C and absorbance was measured at 450 nm wavelength. The obtained values were normalized to the protein concentration in the lysates assessed by BCA assay.

### Assessment of mitochondrial respiration

Mitochondrial respiration was assessed by measuring the oxygen consumption rate (OCR) using the XFp Extracellular Flux Analyzer (Seahorse Bioscience, North Billerica, MA, USA). Cells were seeded in duplicates at 6 000 cells/well onto XFp miniplates 24 h post transfection with siRNAs or overexpression plasmids. On the following day at 48 h post transfection, cells were washed, and the medium changed to either XF Base medium (Seahorse Bioscience) with 1 mM sodium pyruvate, 2 mM L-glutamine, and 10 mM glucose or to XF Base medium with 2 mM sodium pyruvate with or without 200 mM sodium citrate (Sigma-Aldrich). After 30 min incubation at 37 °C, the cell plates were loaded into the Flux Analyzer for the assay, 1 µM oligomycin, 0.5 µM FCCP, and 0.5 µM rotenone/antimycin A were injected subsequently and three OCR measurements were taken after each injection.

### Generation of inducible shRNA-expressing cell lines

Constructs expressing shRNAs targeting USP39 were produced by cloning USP39 oligomers (shRNA 1: GATTTGGAAGAGGCGAGATAA, shRNA 2: GTTGCCTCCATATCTAATCTT) into the Tet-pLKO-puro plasmid (gift from Dmitri Wiederschain (Addgene plasmid # 21915; http://n2t.net/addgene:21915; RRID:Addgene_21915) [53]) following the previously described procedure [53, 54]. To produce lentiviral vectors, HEK293T cells were transfected with the shRNA constructs together with psPAX2 (gift from Didier Trono (Addgene plasmid # 12260; http://n2t.net/addgene:12260; RRID:Addgene_12260)), and pMD2.G (gift from Didier Trono (Addgene plasmid # 12259; http://n2t.net/addgene:12259; RRID:Addgene_12259)) in a 2.5:1.5:1 ratio using the Lipofectamine^TM^ 2000 transfection reagent (Invitrogen, Thermo Fisher, Waltham, MA, USA). A plasmid with a non-targeting randomized shRNA sequence served as control (gift from Roland Friedel (Addgene plasmid # 98398; http://n2t.net/addgene:98398; RRID:Addgene_98398) [55]). Lentiviral particles were collected after 48 and 72 h, spun down, filtered, and stored at −80 °C in single-use vials. NCI-H1975 cells were transduced with the lentiviral particles in the presence of 12 µg/mL polybrene (Sigma-Aldrich) for 24 h. Afterwards, cells were washed and selected with 1.5 µg/mL puromycin (Sigma-Aldrich) for 14 days to generate stable shRNA-expressing cell lines. Cells were treated with 20 ng/mL doxycycline (Sigma-Aldrich) for 48 h to induce the expression of the shRNAs.

### Cell proliferation and clonogenicity

After doxycycline treatment for 48 h to induce the USP39 knockdown, cells were reseeded in triplicates onto 96-well plates at 1000 cells/well. For the citrate supplementation experiment, medium was changed at the time of reseeding to cell culturing medium supplemented with 200 mM sodium citrate. Cell growth was measured over the subsequent 72 h using the CellTiter-Glo® luminescent cell viability assay (Promega). The luminescent signal was detected on a GloMax Discover Microplate Reader (Promega). Values were normalized to the initial measurement.

Knockdown was induced with doxycycline for 48 h, after which a total of 1000 cells/well were plated onto 12-well plates. Medium was changed every 72–96 h for about 7–9 days. Colonies were washed with PBS, fixed with 1% paraformaldehyde (Sigma-Aldrich), and stained with 0.1% crystal violet (Sigma-Aldrich). Colonies were quantified by dissolving each well with 200 µL 1 M NaOH, transferring 2 × 75 µL into a black 96-well plate, and neutralizing the solution with 1 M HCl. Fluorescence signals were detected using the GloMax Discover Microplate Reader, with an excitation wavelength of 520 nm and emission wavelength of 580–640 nm. Background values were subtracted from each measurement, and the results normalized to the shRNA control.

### Animal studies

All animal studies were approved by the Stockholm Animal Ethics Committee and performed according to the Swedish animal welfare legislation (Ethical permit No. 1185-2022). For the tumor growth study, 18 mice were randomly distributed into 3 groups. Before injection, the stable shRNA-expressing NCI-H1975 cell lines were treated with 20 ng/mL doxycycline for 48 h. 3 × 10^6^ cells in 100 µL of 1:1 PBS and Matrigel (Corning Inc., Corning, NY, USA) were injected into the flanks of female 4–5 week old immunodeficient Athymic nude mice (Rj:ATHYM-Foxn1nu/nu*, purchased from Janvier Labs, Le Genest-Saint-Isle, France). The mice were housed in special pathogen-free conditions and had access to doxycycline-containing drinking water supplemented with 5% sucrose (Sigma-Aldrich) during the entire course of the experiment. Tumor measurements were taken every third day, and the mice were sacrificed on the 19th day post injection. The tumors were carefully removed, their weight measured and then the tumors were snap-frozen in liquid nitrogen and stored at −80 °C until further use. The USP39 knockdown efficiency was determined by qPCR. The tumor volume was calculated by 2 *W* × *L*/2 (*L* = length and *W* = the perpendicular width of the tumor, *L*> *W*).

### Statistical analysis

The data is presented as mean ± SD. Student’s *t*-test (two-sided) was used to compare two groups and calculate *p*-values. *p* < 0.05 was considered significant. The graphs were designed, and the statistics calculated using the GraphPad Prism Software version 9.0.0 (Dotmatics, Boston, MA, USA) or R version 4.2.0.

## Supplementary information

Supplementary Figure 1

Supplementary Table 1

Supplementary Table 2

Supplementary_Figure and table legends

Supplementary Figure 2 Full Western blots

## Data Availability

The datasets generated during and/or analyzed during the current study are available from the corresponding author on reasonable request.
