# Supplementary figures and images for: USP39 regulates pyruvate handling in non-small cell lung cancer
Source: Cell Death Discov. 2024 Dec 18;10:502. doi: 10.1038/s41420-024-02264-0 (PMC11655846; doi:10.1038/s41420-024-02264-0)

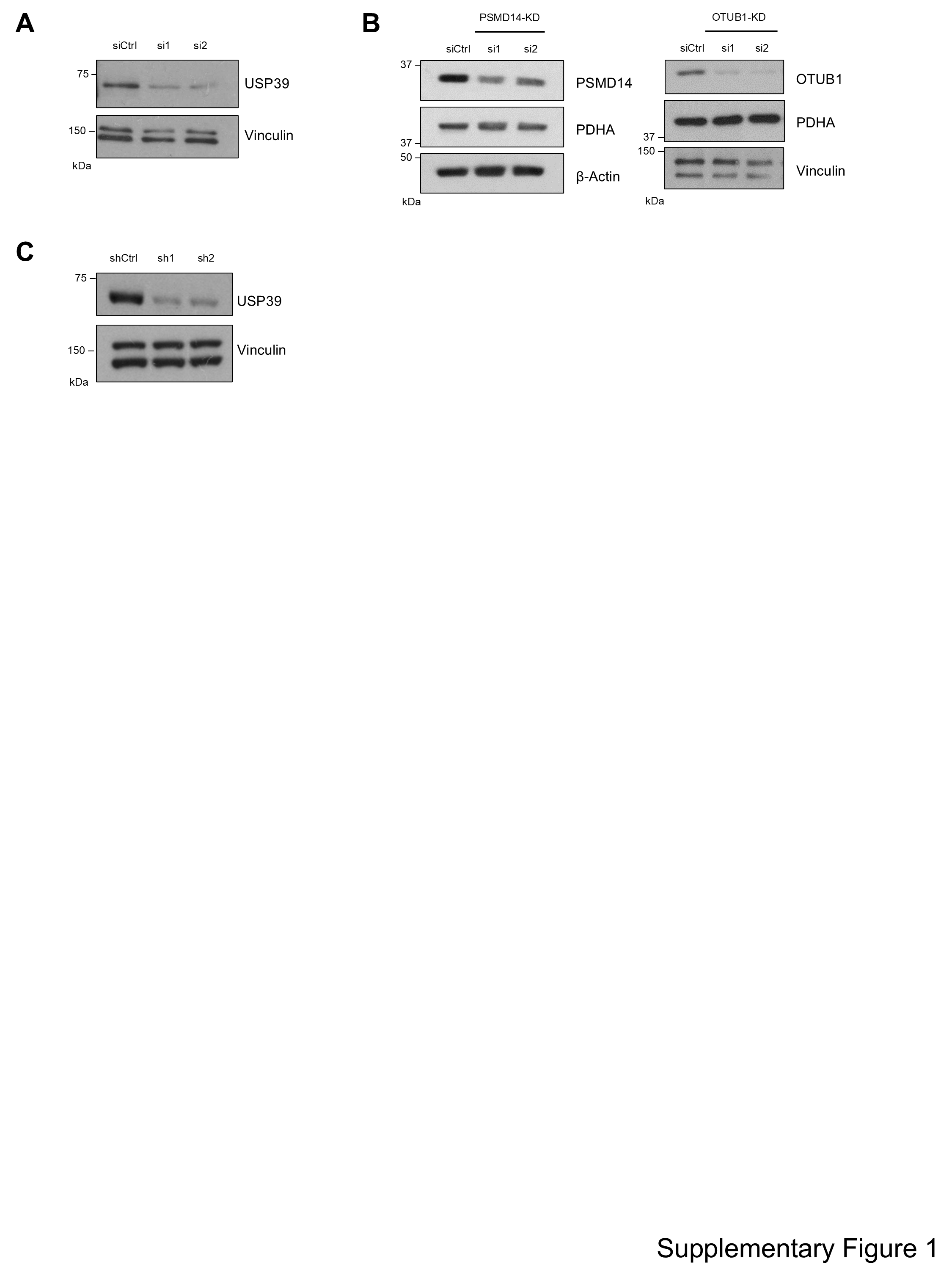

Supplement: Supplementary file 1 — Supplementary Figure 1 [file 41420_2024_2264_MOESM1_ESM.tif]
