# Supplementary material for: USP39 regulates pyruvate handling in non-small cell lung cancer
Source: Cell Death Discov. 2024 Dec 18;10:502. doi: 10.1038/s41420-024-02264-0 (PMC11655846; doi:10.1038/s41420-024-02264-0)
Supplement: Supplementary file 4 — Supplementary_Figure and table legends [file 41420_2024_2264_MOESM4_ESM.docx]

**Supplementary Fig 1:**

**A** USP39 knockdown efficiency after 48 h by two independent siRNAs compared to a non-targeting control (siCtrl). **B** PDHA protein levels after 48 h of siRNA-mediated knockdown of two further DUBs, namely PSMD14 and OTUB1. **C** USP39 knockdown efficiency of stable shRNA-expressing NCI-H1975 cells after 48 h of doxycycline treatment compared to a non-targeting control (shCtrl).

**Supplementary Table 1**

Results of metabolite set enrichment analysis from metabolite levels measured in USP39-deficient cells compared to cells expressing the non-targeting control. Only pathways with a significant FDR (<0.05) are listed in the table.

**Supplementary Table 2**

USP39-associated proteins investigated by co-immunoprecipitation followed by LC-MS/MS analysis. Proteins with at least 3 detected unique peptides in the USP39 immunoconjugates are listed in the table.
