# Supplementary material for: USP39 regulates pyruvate handling in non-small cell lung cancer
Source: Cell Death Discov. 2024 Dec 18;10:502. doi: 10.1038/s41420-024-02264-0 (PMC11655846; doi:10.1038/s41420-024-02264-0)

Figure 2B

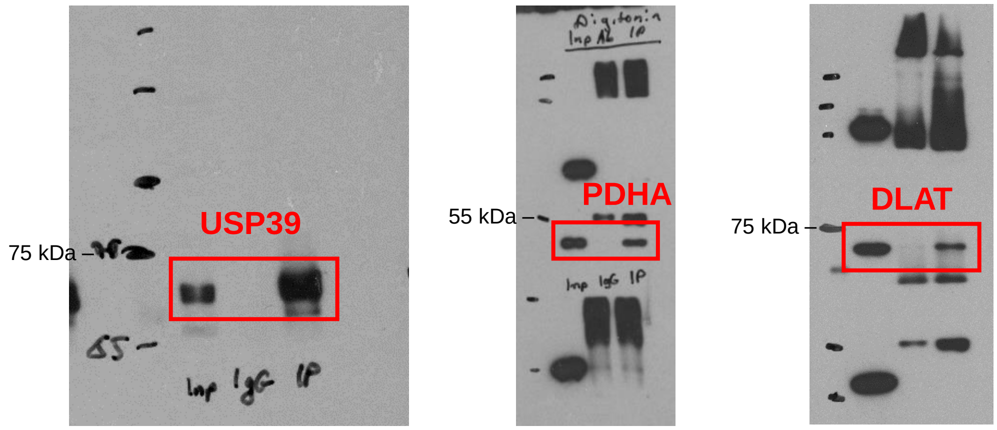

Figure 2C

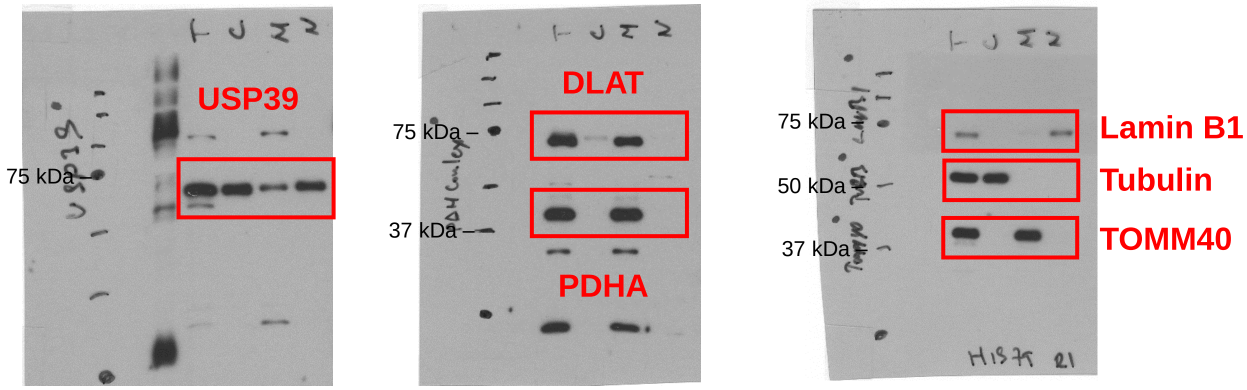

Figure 2D

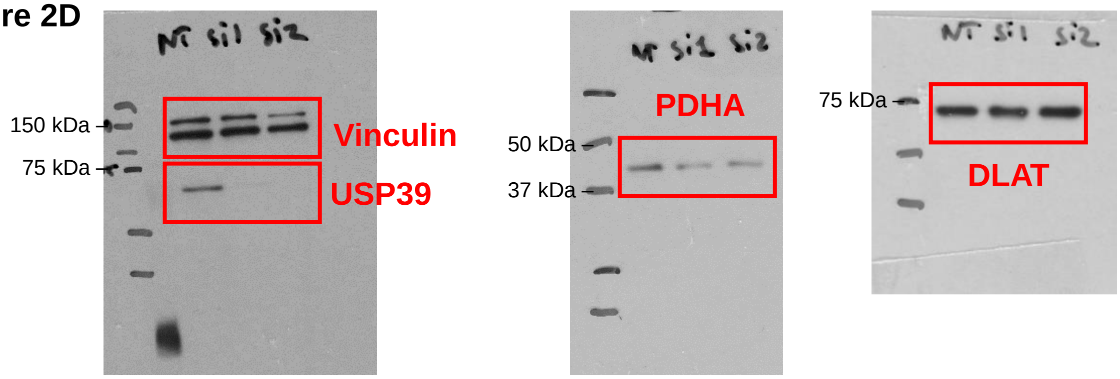

Figure 2E

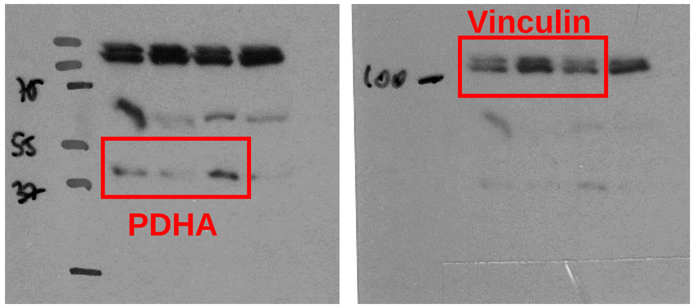

Figure 2H

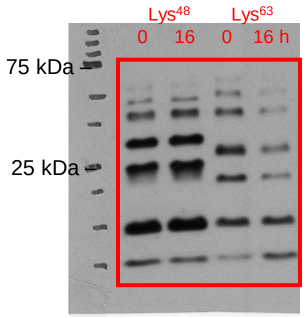

Figure 2I

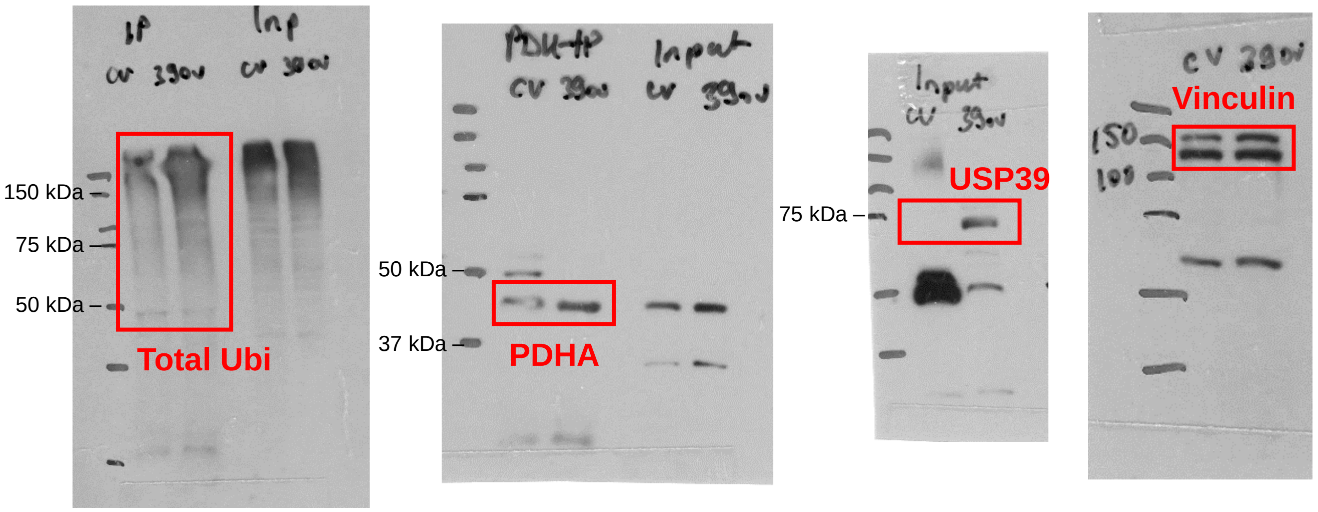

Figure 2J

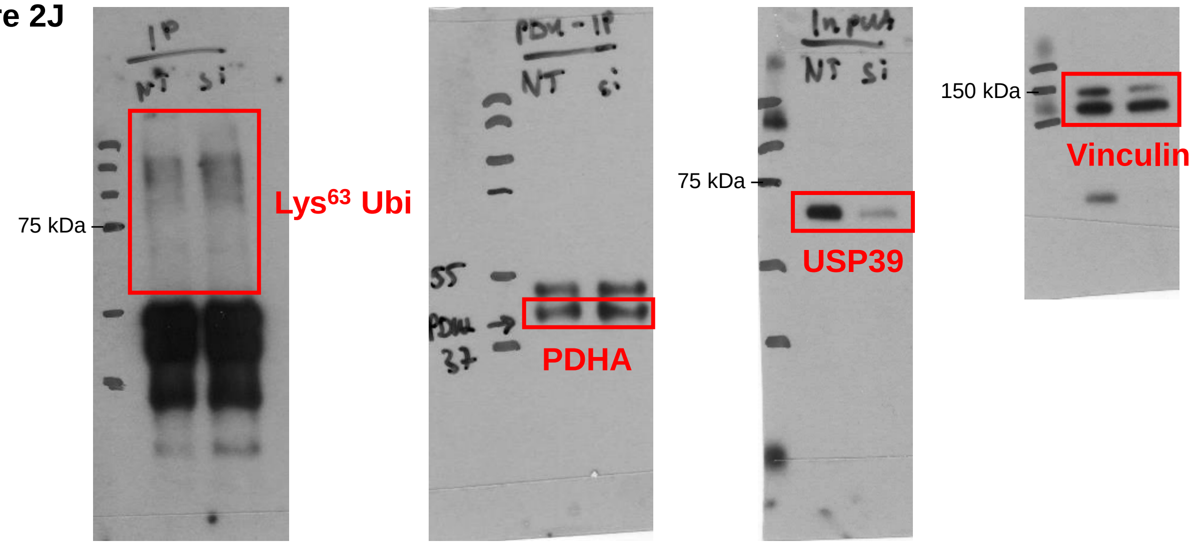

Figure 2K

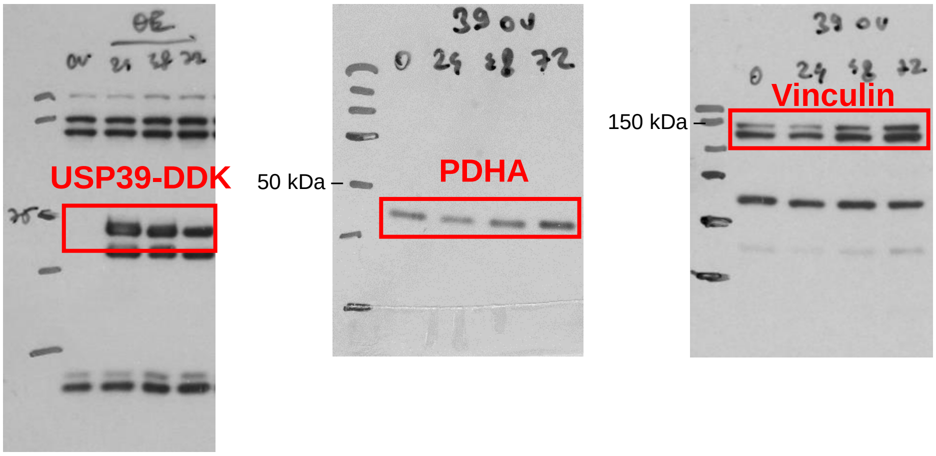

Supplementary Figure 1A

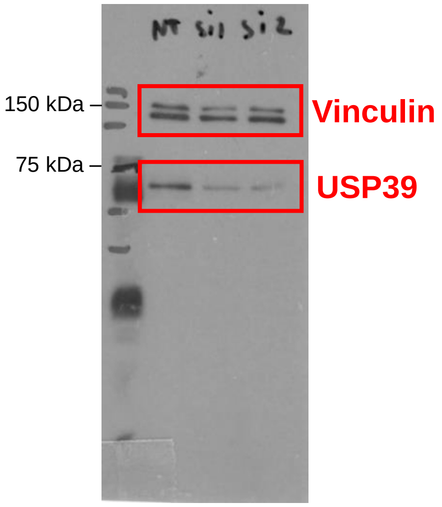

Supplementary Figure 1B

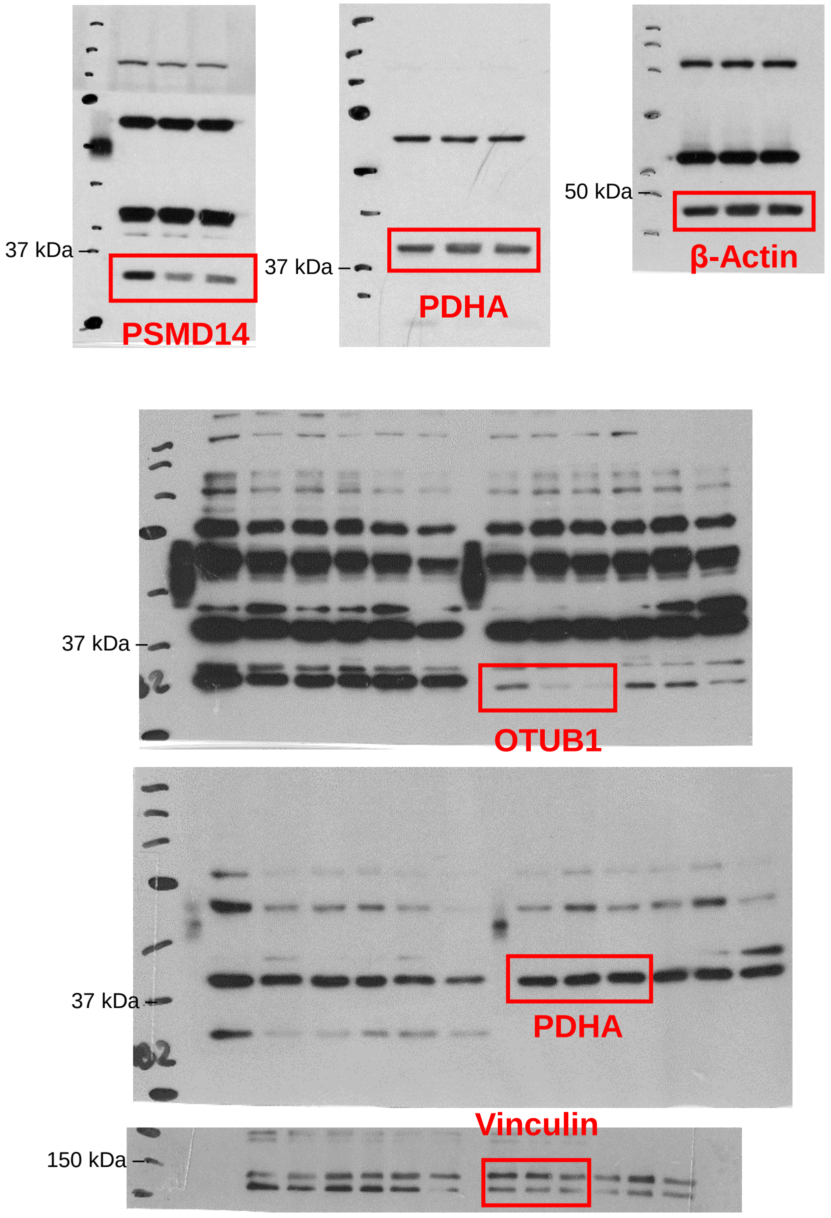

Supplementary Figure 1C

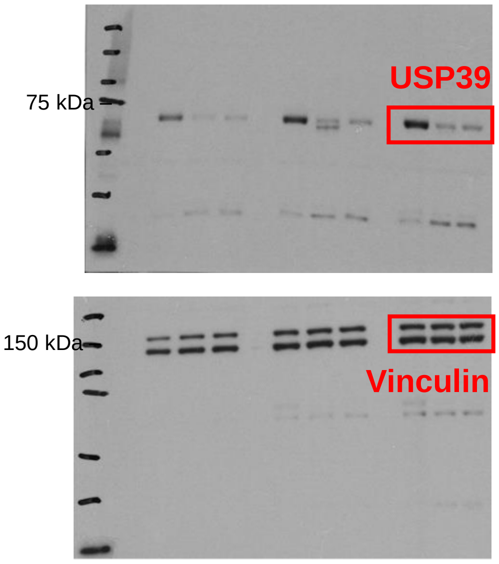

Supplement: Supplementary file 5 — Supplementary Figure 2 Full Western blots [file 41420_2024_2264_MOESM5_ESM.pdf]
